# Hypopyon sign as an unusual complication of varicella infection in a girl with atopic dermatitis

**DOI:** 10.1007/s10354-020-00790-x

**Published:** 2020-12-10

**Authors:** Amélie Gorris, Doris Weiss, Hubert Kogler, Zsolt Szépfalusi, Franz Karlhofer, Alessandra Handisurya, Wolfgang Weninger, Tamar Kinaciyan

**Affiliations:** 1 Department of Dermatology, Medical University of Vienna, Waehringer Guertel 18–20, 1090 Vienna, Austria; 2 St. Anna Children’s Hospital, Vienna, Austria; 3 Department of Pediatrics and Adolescent Medicine, Medical University of Vienna, Vienna, Austria

**Keywords:** Varicella, Bullous impetigo, Hypopyon sign, *Staphylococcus aureus *superinfection, Atopic dermatitis, Varizellen, Bullöse Impetigo, Hypopyonbildung, *Staphylococcus-aureus*-Superinfektion, Atopische Dermatitis

## Abstract

Varicella-zoster virus (VZV) infection, also known as chickenpox, is a common childhood affliction. Generalized small itchy single-standing vesicles on erythematous skin are typical. Both cutaneous and systemic complications of the VZV infection may commonly occur. A three-year-old girl with a previous history of mild atopic dermatitis presented in our Pediatric Dermatology Clinic in poor general condition, with a skin rash predominantly consisting of generalized large blisters with hypopyon sign and erosions. On a closer look, scattered erythematous papules and vesicles were also visible. A positive Tzanck smear from an intact pinhead-sized vesicle and VZV PCR confirmed the clinical diagnosis of chickenpox. Cultures from hypopyon material revealed *Staphylococcus aureus* superinfection. We report an exceptional, not-yet described complication of chickenpox with hypopyon-forming superinfection in an atopic child. In addition, our case nicely underscores the necessity of early VZV vaccination, which has been available and recommended now for more than 10 years in pediatric vaccination programs to avoid severe complications.

## Abstract (de)

Die Varicella-Zoster-Virus(VZV)-Infektion, auch Windpocken genannt, ist eine häufige Kinderkrankheit. Typisch sind generalisierte kleine juckende einzeln stehende Vesikel auf erythematösem Grund. Sowohl kutane als auch systemische Komplikationen treten häufig auf. Ein 3‑jähriges Mädchen mit milder atopischer Dermatitis in der Vorgeschichte stellte sich in der Kinderdermatologie-Ambulanz der Universitätsklinik für Dermatologie Wien mit generalisierten großen Blasen mit Hypopyonbildung und Erosionen vor und war in einem reduzierten Allgemeinzustand. Bei näherer Betrachtung waren zusätzlich disseminierte stecknadelkopfgroße erythematöse Papeln und Vesikel sichtbar. Ein positiver Tzanck-Test von einem intakten stecknadelkopfgroßen Vesikel sowie die VZV-PCR (Polymerasekettenreaktion) bestätigten die klinische Diagnose von Windpocken. Kulturen aus dem Hypopyon-Inhalt zeigten eine Superinfektion mit *Staphylococcus aureus*. Wir berichten hier über eine außergewöhnliche, noch nicht beschriebene Komplikation von Windpocken mit einer hypopyonbildenden Superinfektion bei einem Kind mit atopischer Dermatitis. Darüber hinaus unterstreicht der vorliegende Fall die Notwendigkeit einer frühen VZV-Impfung, die seit mehr als 10 Jahren verfügbar ist und in pädiatrischen Impfprogrammen empfohlen wird, um schwerwiegende Komplikationen zu vermeiden.

## Introduction

Varicella-zoster virus (VZV) is the causative agent of varicella (chickenpox) as primary infection and herpes zoster (shingles) as re-activation of the virus [1]. The primary infection is usually transmitted by inhalation of airborne droplets exhaled from infected hosts. Chickenpox is largely a childhood disease and still very common in Austria. The most common complication in children is bacterial superinfection of skin and soft tissue due to itching arising from varicella.

Atopic dermatitis (AD) is a chronic pruritic inflammatory skin disease, typically starting in early infancy in genetically predisposed persons [2]. In patients suffering from AD, the skin is often colonized by *S. aureus* [2]; this additionally may lead to *S. aureus* superinfections and AD deterioration [3].

## Case report

**Fig. 1:**
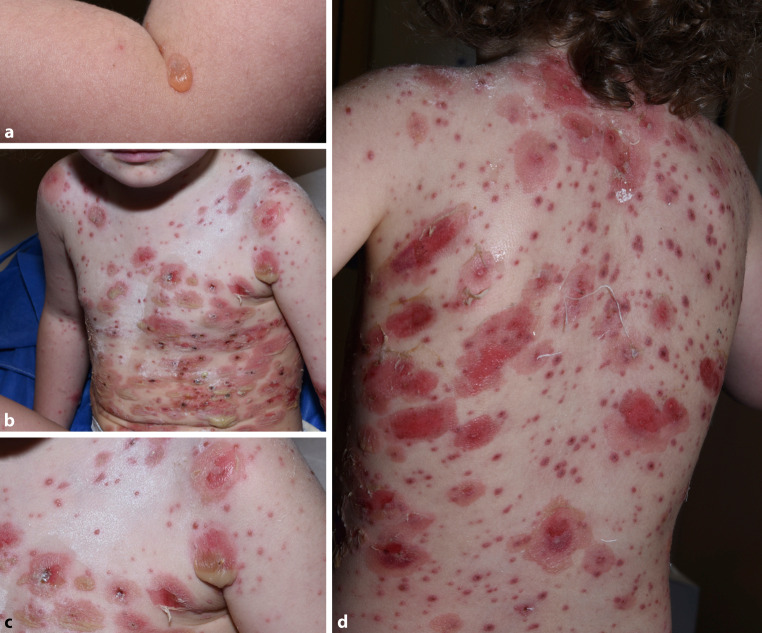
**a** Single plump blister on intact skin as the primary lesion; **b–d** generalized blisters 2–5 cm in diameter, with pus accumulation in the dependent parts (hypopyon sign), erosions at sites of ruptured blisters, and pinhead-sized papules, vesicles, pustules, and crusts surrounded by erythematous skin disseminated between the large blisters

A three-year-old, otherwise healthy girl suffering from mild atopic dermatitis (AD) (SCORAD index of <25) presented in our Pediatric Dermatology Outpatient Clinic in reduced general condition with high-grade fever and a new skin rash. Her skin exhibited generalized blisters of 2–5 cm in diameter, partly translucent, partly with pus accumulation in the dependent part of the flaccid bullae forming a transverse fluid level (hypopyon sign) [3, 4]. Erosions were visible at sites of ruptured blisters (Fig. 1b–d). Only a single plump blister on intact skin of the left arm was visible as the primary lesion (Fig. 1a). Further inspection revealed pinhead-sized papules, vesicles, pustules, erosions, and crusts surrounded by erythematous skin disseminated between the large blisters (Fig. 1b–d). Palms, soles, scalp as well as genital and oral mucosa were disease free.

Rapid diagnosis was made by Tzanck smear from a small intact vesicle revealing multinucleated giant cells and acantholytic keratinocytes. Herpes polymerase chain reaction (PCR) from both serum and blisters were positive for varicella-zoster virus (VZV). VZV serology demonstrated a recent infection (IgM antibodies positive, IgG antibodies negative). In addition, bacterial culture from the skin smear revealed superinfection with *Staphylococcus aureus *without methicillin resistance.

**Fig. 2:**
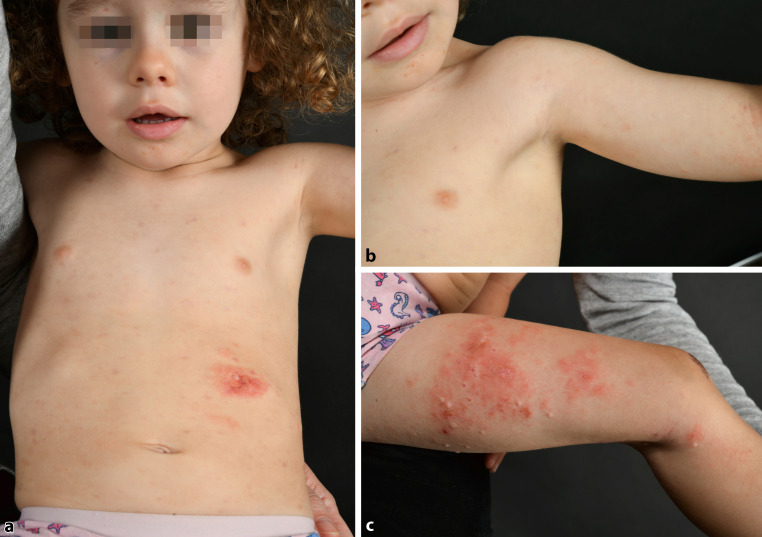
**a–c** *S.* *aureus* and varicella-zoster virus infection healed without any scars and residue revealing some eczema plaques; **c** Mollusca contagiosa, another atopic dermatitis-associated viral superinfection, on the legs that were masked before

Immediately after diagnosing chickenpox in the non-VZV-vaccinated girl combined with bullous impetigo and hypopyon sign due to *Staphylococcus aureus* superinfection on the basis of atopic skin and VZV infection, the patient was isolated in the pediatric ward and received acyclovir 130 mg TID (10 mg/kg/day) and amoxicillin/clavulanic acid 650 mg TID (50 mg/kg/day) intravenously for 5 days. She also received parenteral fluid substitution, paracetamol infusions, and oral antihistamines. Subsequently, treatment was switched to oral therapy for an additional 5 days. She was discharged after 10 days. The topical treatment consisted of neomycin sulfate powder on intact blisters and methylprednisolone aceponate 0.1% cream plus sterile paraffin gauze dressing of the erosive skin. She recovered completely, only some eczema plaques persisted from the known AD (Fig. 2a–c).

## Discussion

VZV infection is a common, airborne droplet-transmitted primary, highly contagious, acute childhood infection with typical morphology and distribution of skin lesions—easy to diagnose for the trained clinician [1]. While the prognosis is generally good, hospitalization rate is about 6 out of 100,000 children aged 0–15 years [5]. The most common complication in 31 to 70% of all varicella complications is bacterial superinfection, and superficial skin infections account for 20 to 50% [6]. Massive bacterial infection can make the diagnosis of VZV infection challenging and lead to the disorder to be overlooked. Bacterial superinfections are facilitated by skin barrier disruption due to itching and skin excoriation and possibly by transient virus-induced alterations of local immunity followed by staphylococcal infection syndromes [6]. Raulin et al. analyzed the profile of *S. aureus-*specific toxins in varicella superinfections and highlighted that severe forms are mostly related to methicillin-resistant *Staphylococcus aureus* (MRSA) strains [6]. In our patient, smear cultures where fortunately negative for MRSA.

Hypopyon sign is rarely reported in secondarily infected vesiculobullous autoimmune disorders [4], but has not yet been in varicella infection or in atopic dermatitis in children.

In several European countries and the United States, VZV vaccination is a fixed component of vaccination programs for children between the 12th and 18th month of life [5]. Since 2010, the Austrian vaccination program strongly recommends and provides VZV vaccination for children at the beginning of the second year, which represents the best prevention of infection-associated complications; however, its implementation is still very inconsistent. A change in the incidence of the infection and its complications can only be expected after nationwide vaccination [7, 8].

## Conclusion

The peculiarity of our case is that i) the typical distribution of chickenpox was missing, as palms, soles, scalp as well as oral mucosa were disease free; ii) predominant large blisters and unusual hypopyon sign almost masked the acute VZV infection, leading to the disorder being overlooked; iii) although bacterial superinfections are common in chickenpox, hypopyon sign has, to the best of our knowledge, not yet been reported. In addition, our case nicely underscores the necessity of early VZV vaccination in children to avoid severe complications and hospitalization.
